# Supplementary material for: Outcomes of asymptomatic recombinant tissue plasminogen activator associated intracranial hemorrhage
Source: PLoS One. 2022 Aug 1;17(8):e0272257. doi: 10.1371/journal.pone.0272257 (PMC9342748; doi:10.1371/journal.pone.0272257)
Supplement: Attachment — Submitted filename: EVALUATION.docx [file pone.0272257.s001.docx]

EVALUATION

1) First of all, I would like to thank you for the article. In line 164-165, ‘’There were no 165 significant differences of the baseline characteristics of patients with regards to sex, age, comorbid diseases and prior medication use’’, How was this conclusion reached? Maybe the author should be more clear. Thank you for your efforts.
2) Points should be after the references.
3) Some English words should be checked .(125: ~~past~~ medical history )
