# Supplementary material for: Outcomes of asymptomatic recombinant tissue plasminogen activator associated intracranial hemorrhage
Source: PLoS One. 2022 Aug 1;17(8):e0272257. doi: 10.1371/journal.pone.0272257 (PMC9342748; doi:10.1371/journal.pone.0272257)
Supplement: Attachment — Submitted filename: Response to Reviewers.docx [file pone.0272257.s002.docx]

**Reviewer Comments, Responses, and Manuscript Changes**

**Reviewer 1**

1. **In lines 164-165, There were not 165 significant differences of the baseline characteristics of patients with regards to sex, age, comorbid diseases, and prior medication use, How was this conclusion reached?**

**Response:**

We thank the reviewer for the comment. We performed the univariable analyses comparing the differences among three groups (no ICH vs asymptomatic intracranial hemorrhage (aICH), no ICH vs symptomatic intracranial hemorrhage (sICH), and aICH vs sICH). When comparing three groups (using ANOVA or crosstab function), there was no statistical difference of variables. However, some variables had statistically significant differences between groups. Thus, we revised and added the information in Table 1.

**Table 1. Baseline Characteristic of Patients Who Received Recombinant Tissue Plasminogen Activator for Acute Ischemic Stroke**

| Characteristics/factors | No ICH  (n=559) | aICH (n=116) | sICH (n=50) | P-value | | |
| --- | --- | --- | --- | --- | --- | --- |
|  |  |  |  | No ICH vs aICH | No ICH va sICH | aICH vs sICH |
| Male sex — no. (%) | 288 (51.5) | 51 (44.0) | 25 (50.0) | 0.14 | 0.84 | 0.47 |
| Age — median (IQR) | 65.0 (55.0, 75.0) | 67.0 (58.0, 76.0) | 70.5 (63.0, 74.0) | 0.37 | 0.87 | 0.63 |
| Weight (kg) — median (IQR) | 58.0 (50.7, 67.0) | 60.5 (50.0, 67.5) | 54.5 (48.0, 64.1) | 0.55 | 0.09 | 0.06 |
| Co-morbid diseases — no. (%) | | | | | | |
| Hypertension | 356 (63.7) | 82 (70.7) | 33 (66.0) | 0.15 | 0.74 | 0.55 |
| Diabete mellitus | 109 (19.5) | 27 (23.3) | 17 (34.0) | 0.36 | 0.02† | 0.15 |
| Atrial fibrillation | 67 (12.0) | 32 (27.6) | 19 (38.0) | <0.001† | <0.001† | 0.18 |
| Dyslipidemia | 170 (30.4) | 37 (31.9) | 14 (28.0) | 0.75 | 0.72 | 0.62 |
| Congestive heart failure | 39 (7.0) | 7 (6.0) | 7 (14.0) | 0.71 | 0.07 | 0.09 |
| Coronary artery disease | 52 (9.3) | 17 (14.7) | 6 (12.0) | 0.08 | 0.53 | 0.65 |
| Peripheral arterial disease | 2 (0.4) | 1 (0.9) | 0 (0.0) | 0.46 | 0.67 | 0.51 |
| Recurrent stroke | 44 (7.9) | 7 (6.0) | 2 (4.0) | 0.50 | 0.32 | 0.60 |
| Smoking | 79 (14.1) | 12 (10.3) | 8 (16.0) | 0.28 | 0.72 | 0.30 |
| Alcohol consumption | 57 (10.2) | 14 (12.1) | 9 (18.0) | 0.55 | 0.09 | 0.31 |
| Antiplatelet/anticoagulant — no. (%) | | | | | | |
| Anticoagulant | 33 (5.9) | 10 (8.6) | 3 (6.0) | 0.28 | 0.98 | 0.56 |
| Antiplatelet | 91 (16.3) | 31 (26.7) | 18 (36.0) | 0.01† | <0.001† | 0.23 |
| TOAST classification — no. (%) | | | | | | |
| Atherosclerosis | 331 (59.2) | 36 (31.0) | 17 (34.0) | <0.001† | 0.001† | 0.71 |
| Cardioembolism | 150 (26.8) | 70 (60.3) | 31 (62.0) | <0.001† | <0.001† | 0.84 |
| Small vessel diseases | 48 (8.6) | 5 (4.3) | 1 (2.0) | 0.12 | 0.10 | 0.46 |
| Other etiology | 17 (3.0) | 5 (4.3) | 1 (2.0) | 0.48 | 0.68 | 0.46 |
| Undetermined | 13 (2.3) | 0 (0.0) | 0 (0.0) | 0.10 | 0.28 | - |
| Admission profiles — median (IQR) | | | | | | |
| Onset to needle (minutes) | 159.0 (122.0, 200.0) | 154.5 (120.0, 186.0) | 150,0 (120.0, 210.0) | 0.15 | 0.52 | 0.78 |
| Door to needle (minutes) | 55.0 (45.0, 71.0) | 55.0 (46.0, 66.0) | 50.0 (44.0, 66.0) | 0.15 | 0.82 | 0.48 |
| Admission NIHSS — median (IQR) | 9 (5, 15) | 13 (8, 18) | 17 (12, 21) | 0.05† | <0.001† | 0.21 |
| Admission Barthel index — median (IQR) | 25 (10, 55) | 20 (5, 40) | 10 (0, 15) | 0.03† | 0.01† | 0.22 |
| Admission mRS >2 — no. (%) | 514 (92.0) | 112 (96.6) | 49 (98.0) | 0.08 | 0.12 | 0.62 |

Abbreviations: ICH, intracranial hemorrhage; aICH, asymptomatic intracranial hemorrhage; sICH, symptomatic intracranial hemorrhage; rtPA, recombinant tissue plasminogen activator; kg, kilogram; TOAST, the trial of ORG 10172 in acute stroke treatment; IQR, interquartile range; NIHSS, National Institute of Health Stroke Scale; mRS, modified Rankin Scale; no., number

† Statistically significant

We revised the paragraph on page 8, line 163:
from “*There were no significant differences of the baseline characteristics of patients with regards to sex, age, co-morbid diseases, and prior medication use*.”
To “*Table 1 demonstrates baseline clinical characteristics of patients, classified into three groups (no ICH, aICH, and sICH). Atrial fibrillation and prior antiplatelet use were less frequently found in the no ICH group comparing to aICH and sICH groups. As compared to the no ICH group, the aICH and sICH groups had more unfavorable admission characteristics (higher mRS, higher NIHSS and lower Barthel index).*”

2. **Points should be after the references.**

**Response** We revised the entire manuscript as suggested.

3. **Some English words should be checked. (125: ~~past~~ medical history** )

**Response** We revised the sentence as suggested on page 6, line 125:

from “*We collected demographic data, past medical history, laboratory data, and imaging data at stroke diagnosis*.”

to “*We collected demographic data, medical history, laboratory data, and imaging data at stroke diagnosis*.”

**Reviewer 2**

1. **I suggest paying attention to discuss the difference between patients with aICH and without ICH, especially regarding the risk factors (history, stroke classification, admission BP, sugar, stroke size, etc), potential predictors of aICH compared with patients without ICH, management (whether most patients with aICH did not need aggressive treatment) and outcome comparison. It may be an issue that is less discussed in previous literature. So the data presentation may be considered to be revised.**

**Response:**

1) We thank the reviewer for these valuable comments. We revised Table 1. We compared the baseline characteristics of patients who did not have ICH and asymptomatic ICH. We performed the univariable analyses to compare the differences between the no ICH group and aICH group. Atrial fibrillation, prior antiplatelet use, and cardioembolic stroke subtype according to TOAST classification were less frequent in the no ICH group comparing to aICH group. On the other hand, the atherosclerotic stroke subtype was more frequently observed in the no ICH group than aICH group. Initial presentation of patients in the no ICH group was more favorable compared to aICH (lower admission NIHSS (median 9 [IQR 5, 15] vs 13 [IQR 8, 18], P-value =0.05) and higher Barthel index (median 25 [IQR 10, 55] vs 20 [IQR 5, 40], P-value =0.03)), respectively.

We revised the paragraph on page 8, line 163:

from “*There were no significant differences of the baseline characteristics of patients with regards to sex, age, co-morbid diseases, and prior medication use*.”

To “*Table 1 demonstrates baseline clinical characteristics of patients, classified into three groups (no ICH, aICH, and sICH). Atrial fibrillation and prior antiplatelet use were less frequently found in the no ICH group comparing to aICH and sICH groups. As compared to the no ICH group, the aICH and sICH groups had more unfavorable admission characteristics (higher mRS, higher NIHSS and lower Barthel index).*”

We added a new sentence on page 8, line 164:

*“Atrial fibrillation and prior antiplatelet used were less frequently found in no ICH group comparing to aICH and sICH groups.”*

We added the paragraph on page 9, line 168:

*“When comparing between patients who did not have ICH and those with aICH, the no ICH group had more atherosclerotic and less cardioembolic subtypes per the TOAST classification. Initial presentation of patients in no ICH group was more favorable compared to aICH (lower admission NIHSS (median 9 [IQR 5, 15] vs 13 [IQR 8, 18], P-value =0.05) and higher Barthel index (median 25 [IQR 10, 55] vs 20 [IQR 5, 40], P-value =0.03)), respectively.”*

We revised Table 1.

**Table 1. Baseline Characteristic of Patients Who Received Recombinant Tissue Plasminogen Activator for Acute Ischemic Stroke**

| Characteristics/factors | No ICH  (n=559) | aICH (n=116) | sICH (n=50) | P-value | | |
| --- | --- | --- | --- | --- | --- | --- |
|  |  |  |  | No ICH vs aICH | No ICH va sICH | aICH vs sICH |
| Male sex — no. (%) | 288 (51.5) | 51 (44.0) | 25 (50.0) | 0.14 | 0.84 | 0.47 |
| Age — median (IQR) | 65.0 (55.0, 75.0) | 67.0 (58.0, 76.0) | 70.5 (63.0, 74.0) | 0.37 | 0.87 | 0.63 |
| Weight (kg) — median (IQR) | 58.0 (50.7, 67.0) | 60.5 (50.0, 67.5) | 54.5 (48.0, 64.1) | 0.55 | 0.09 | 0.06 |
| Co-morbid diseases — no. (%) | | | | | | |
| Hypertension | 356 (63.7) | 82 (70.7) | 33 (66.0) | 0.15 | 0.74 | 0.55 |
| Diabete mellitus | 109 (19.5) | 27 (23.3) | 17 (34.0) | 0.36 | 0.02† | 0.15 |
| Atrial fibrillation | 67 (12.0) | 32 (27.6) | 19 (38.0) | <0.001† | <0.001† | 0.18 |
| Dyslipidemia | 170 (30.4) | 37 (31.9) | 14 (28.0) | 0.75 | 0.72 | 0.62 |
| Congestive heart failure | 39 (7.0) | 7 (6.0) | 7 (14.0) | 0.71 | 0.07 | 0.09 |
| Coronary artery disease | 52 (9.3) | 17 (14.7) | 6 (12.0) | 0.08 | 0.53 | 0.65 |
| Peripheral arterial disease | 2 (0.4) | 1 (0.9) | 0 (0.0) | 0.46 | 0.67 | 0.51 |
| Recurrent stroke | 44 (7.9) | 7 (6.0) | 2 (4.0) | 0.50 | 0.32 | 0.60 |
| Smoking | 79 (14.1) | 12 (10.3) | 8 (16.0) | 0.28 | 0.72 | 0.30 |
| Alcohol consumption | 57 (10.2) | 14 (12.1) | 9 (18.0) | 0.55 | 0.09 | 0.31 |
| Antiplatelet/anticoagulant — no. (%) | | | | | | |
| Anticoagulant | 33 (5.9) | 10 (8.6) | 3 (6.0) | 0.28 | 0.98 | 0.56 |
| Antiplatelet | 91 (16.3) | 31 (26.7) | 18 (36.0) | 0.01† | <0.001† | 0.23 |
| TOAST classification — no. (%) | | | | | | |
| Atherosclerosis | 331 (59.2) | 36 (31.0) | 17 (34.0) | <0.001† | 0.001† | 0.71 |
| Cardioembolism | 150 (26.8) | 70 (60.3) | 31 (62.0) | <0.001† | <0.001† | 0.84 |
| Small vessel diseases | 48 (8.6) | 5 (4.3) | 1 (2.0) | 0.12 | 0.10 | 0.46 |
| Other etiology | 17 (3.0) | 5 (4.3) | 1 (2.0) | 0.48 | 0.68 | 0.46 |
| Undetermined | 13 (2.3) | 0 (0.0) | 0 (0.0) | 0.10 | 0.28 | - |
| Admission profiles — median (IQR) | | | | | | |
| Onset to needle (minutes) | 159.0 (122.0, 200.0) | 154.5 (120.0, 186.0) | 150,0 (120.0, 210.0) | 0.15 | 0.52 | 0.78 |
| Door to needle (minutes) | 55.0 (45.0, 71.0) | 55.0 (46.0, 66.0) | 50.0 (44.0, 66.0) | 0.15 | 0.82 | 0.48 |
| Admission NIHSS — median (IQR) | 9 (5, 15) | 13 (8, 18) | 17 (12, 21) | 0.05† | <0.001† | 0.21 |
| Admission Barthel index — median (IQR) | 25 (10, 55) | 20 (5, 40) | 10 (0, 15) | 0.03† | 0.01† | 0.22 |
| Admission mRS >2 — no. (%) | 514 (92.0) | 112 (96.6) | 49 (98.0) | 0.08 | 0.12 | 0.62 |

Abbreviations: ICH, intracranial hemorrhage; aICH, asymptomatic intracranial hemorrhage; sICH, symptomatic intracranial hemorrhage; rtPA, recombinant tissue plasminogen activator; kg, kilogram; TOAST, the trial of ORG 10172 in acute stroke treatment; IQR, interquartile range; NIHSS, National Institute of Health Stroke Scale; mRS, modified Rankin Scale; no., number

† Statistically significant

2) Concerning predictors of aICH, we created Table 2 to demonstrate the results of a multivariable analysis of predictors for aICH. Cardioembolic stroke subtype was associated with an increased risk of aICH with an OR of 3.7 (95% CI 2.4-5.7). Moreover, admission NIHSS more than 15 was associated with the increment of aICH, OR 1.9 (95% CI 1.2-3.0)

We added the new topic on page 9, line 173:

*“Risk factors of asymptomatic intracranial hemorrhage”*

We added the paragraph on page 9, line 174

*“Table 2 demonstrates the results of multivariable analysis of risk factors for aICH. Cardioembolic stroke subtype was associated with an increased risk of aICH with an OR of 3.7 (95% CI 2.4-5.7). Moreover, admission NIHSS more than 15 was associated with the increment of aICH, OR 1.9 (95% CI 1.2-3.0)”*

We added Table 2.

**Table 2 Risk Factors of Asymptomatic Intracranial Hemorrhage**

| Risk factor | Odds Ratios (95% Confident Interval) | P-value |
| --- | --- | --- |
| Cardioembolic stroke subtype | 3.7 (2.4-5.7) | <0.001 |
| Admission NIHSS >15 | 1.9 (1.2-3.0) | 0.003 |

Abbreviations: NIHSS, National Institute of Health Stroke Scale
† Statistically significant

3) Concerning management, all patients who did not have ICH did not require blood transfusion or neurological intervention.

We revised the paragraph on page 10, line 191:
*“Table 4 demonstrates the management of rtPA-associated bleeding. The patients with aICH received any type of blood products less than the patients with sICH (7.8% vs 80.0%, P-value <0.001). Fresh frozen plasma was the most common blood product used, followed by cryoprecipitate. Packed red blood cell transfusion was given in 0.9% of the aICH group and 32.0% in the sICH group (P-value <0.001). Craniotomy was performed in 0.9% of the aICH group and 60.0% in the sICH group (P-value <0.001).”*

We added the paragraph on page 10, line 197: *“Focusing on patients without ICH, transfusion of blood product or neurosurgical intervention was not required in all patients.”*

4) Concerning the outcomes, the patients without ICH had more favorable outcomes comparing with the patients with aICH and sICH (higher proportion of mRS >2, NIHSS >15, Barthel <25, and higher mortality rate at day 14 and day 90) as shown in Figure 2, 3 and 4. We analyzed the association of length of stay (LOS) vs no ICH, aICH and sICH groups. We found that the no ICH group had a shorter length of stay (LOS) comparing with aICH group and sICH group (median 5 days (IQR 3, 9), 10 days (IQR 5, 20) and 9 days (IQR 5,15), P-value < 0.001), respectively.

We added the paragraph on page 11, line 208:
*“Furthermore, the no ICH group had a shorter length of stay (LOS) comparing with aICH group and sICH group (median 5 days [IQR 3, 9],10 days [IQR 5, 20] and 9 days [IQR 5, 15], P-value <0.001), respectively.”*

We revised the paragraph on page 11, line 216:

*“NIHSS and Barthel index at 14 and 90 days after stroke diagnosis are shown in Figures 3 and 4. A higher proportion of patients with sICH had the worst stroke outcomes (defined as NIHSS >15 or Barthel index <25) compared to those with no ICH and aICH at day 14 (P-value =0.003 and P-value <0.001, respectively), and at day 90 (P-value <0.001 and P-value <0.001, respectively).”*

We added the new topic on page 11, line 221:

*“Mortality”*

We added the paragraph on page 12, line 222:
*“Patients in the no ICH group had a significantly lower proportion of all-cause mortality at 14 days and 90 days as compared to patients with aICH and sICH. The Kaplan-Meier curve of overall survival is demonstrated in Figure 5. When compared to no ICH patients, aICH and sICH were associated with an increased risk of 90-day mortality adjusted by age, HR 3.7, 95% CI 1.6-8.9, and HR 40.8, 95% CI 20.7-80.6, respectively.”*

We revised Figure 5.

**Figure 5**

2. **I think the statistical method should be revised, especially regarding the functional outcome comparison between groups. Some potential clinical factors should be put into multivariate analysis. From the current manuscript, I can't confirm which factors have been put into multivariate analysis.**

**Response**:

1.) We performed the univariable and multivariable analyses to evaluate the risk factors of the poor outcome as defined by mRS >2. Table 5 demonstrated the independent risk factors for poor stroke outcomes. After adjusting for confounders (age, atrial fibrillation, serum albumin level, admission NIHSS, and Barthel index), aICH and sICH increased risk of unfavorable stroke outcome.

We added the paragraph on page 10, line 203:

*“Table 5 demonstrated multivariable analysis of unfavorable mRS at 90 days. After adjusting for confounders (age, atrial fibrillation, serum albumin level, admission NIHSS, and Barthel index), aICH and sICH increased risk of unfavorable stroke outcome when compared to patients without ICH with OR 2.3 (95% CI 1.5-3.6), P-value <0.001, and OR 21.8 (95% CI 5.1-92.4), P-value <0.001, respectively.”*

We added Table 5

**Table 5** **Multivariable Analysis of Unfavorable Outcomes Defined as Modified Rankin Scale > 2 in Patients Who Had Acute Ischemic Stroke and Received rtPA.**

| Characteristics | Odds Ratios (95% Confident Interval) | P-value |
| --- | --- | --- |
| No ICH  aICH  sICH | Reference  2.3 (1.5-3.6)  21.8 (5.1-92.4) | Reference  <0.001  <0.001 |
| Age | 1.0 (1.0-1.0) | 0.01 |
| Prior atrial fibrillation | 2.4 (1.6-3.7) | <0.001 |
| Recurrent stroke | 2.7 (1.5-4.9) | 0.001 |
| Higher admission NIHSS | 1.1 (1.1-1.1) | <0.001 |
| Higher admission mRS | 1.4 (1.2-1.6) | <0.001 |
| Lower admission Barthel index | 1.0 (1.0-1.0) | <0.001 |
| Lower albumin level | 0.6 (0.4-0.8) | <0.001 |

Abbreviations: rtPA, recombinant tissue plasminogen activator; ICH, intracranial hemorrhage; aICH, asymptomatic intracranial hemorrhage; sICH, symptomatic intracranial hemorrhage; NIHSS, National Institute of Health Stroke Scale; mRS, modified Rankin Scale

2.) Concerning the all-cause mortality. We performed a univariable analysis using the Cox-Proportional hazard model. Table 1R was for review propose only. We found that intracranial hemorrhage was the only clinical risk factor that was independently associated with death. We decided to include age in the final Cox-Proportional hazard model due to the biological plausibility that age might contribute to mortality. Table 1R (for review only) demonstrates the hazard ratios for all-cause mortality in the final model that included ICH status and age. Consequently, we reported the hazard ratios of all-cause mortality in the manuscript page 11, line 225, after adjusting for age.

**Table 1R. (for review) Cox-Proportional Hazard Model for All-Cause Mortality**

| Variables | Hazard Ratios  (95% Confident Interval) | P-value |
| --- | --- | --- |
| No ICH  aICH  sICH | Reference  3.7 (1.6-8.9)  40.8 (20.7-80.6) | Reference  <0.001  <0.001 |
| Age | 1.0 (1.0-1.0) | 0.09 |
| Atrial fibrillation | 1.2 (0.6-2.5) | 0.49 |
| Recurrent Stroke | 1.7 (0.7-4.1) | 0.20 |
| Atherosclerosis subtype | 1.0 (0.6-1.8) | 0.87 |
| Cardioembolic ssubtype | 1.0 (0.5-1.7) | 0.92 |
| Admission NIHSS | 1.0 (1.0-1.0) | 0.96 |
| Admission mRS | 1.0 (0.7-1.1) | 0.58 |
| Admission Barthel index | 1.0 (1.0-1.0) | 0.59 |

Abbreviations: ICH, intracranial hemorrhage; aICH, asymptomatic intracranial hemorrhage; sICH, symptomatic intracranial hemorrhage; NIHSS, National Institute of Health Stroke Scale; mRS, modified Rankin Scale

3. **I think the data presented in the table should be checked. For example, in Table 1, the overall patient number in the column of TOAST classification in patients without ICH (331+150+48+17) is fewer than the original number (559). Such same problem was also noted regarding the number in the column of prior medication.**

**Response**:

We revised the data in Table 1 as demonstrated above.

1) In TOAST classification, we added the “*undetermined*” row.
2) We corrected the percentage of unfavorable outcomes in the no ICH group. On page 10 line 202, we changed from *“40.5%”* to *“25.9%”.*

**Editor Requirement**

1. **Please ensure that your manuscript meets PLOS ONE's style requirements, including those for file naming. The PLOS ONE style templates can be found at**

**Response**: We change the name to “PLOSOne_asymptomaticICH_Teekaput_et_al.pdf”

**2. a) Please clarify the sources of funding (financial or material support) for your study. List the grants or organizations that supported your study, including funding received from your institution. 
b) State what role the funders took in the study. If the funders had no role in your study, please state: “The funders had no role in study design, data collection, and analysis, decision to publish, or preparation of the manuscript.”
c) If any authors received a salary from any of your funders, please state which authors and which funders.
d) If you did not receive any funding for this study, please state: “The authors received no specific funding for this work.”**

**Please include your amended statements within your cover letter; we will change the online submission form on your behalf.**

**Response:** The authors received no specific funding for this work. We stated in the cover letter as required.

**3. Funding: None
We note that you have provided funding information that is not currently declared in your Funding Statement. However, funding information should not appear in the Acknowledgments section or other areas of your manuscript. We will only publish funding information present in the Funding Statement section of the online submission form. 
Please remove any funding-related text from the manuscript and let us know how you would like to update your Funding Statement. Currently, your Funding Statement reads as follows: 
"No"**

**Please include your amended statements within your cover letter; we will change the online submission form on your behalf.**

**Response:** The authors received no specific funding for this work. We remove “Funding: None” from the manuscript but do state it in the cover letter instead.

**4. Thank you for stating the following in your Competing Interests section:  
"NO authors have competing interests". 
Please complete your Competing Interests on the online submission form to state any Competing Interests. If you have no competing interests, please state "The authors have declared that no competing interests exist.", as detailed online in our guide for authors at** [**http://journals.plos.org/plosone/s/submit-now**](http://journals.plos.org/plosone/s/submit-now) 
 **This information should be included in your cover letter; we will change the online submission form on your behalf.**

**Response:** All authors declare no conflict of interest. We remove “Conflict of Interest: All authors declare no conflict of interest” from the manuscript but do state it in the cover letter instead.

**5.We note that you have stated that you will provide repository information for your data at acceptance. Should your manuscript be accepted for publication, we will hold it until you provide the relevant accession numbers or DOIs necessary to access your data. If you wish to make changes to your Data Availability statement, please describe these changes in your cover letter and we will update your Data Availability statement to reflect the information you provide.**

**Response**:

**6.Please ensure that you refer to Figure 5 in your text as, if accepted, production will need this reference to link the reader to the figure.**

**Response:** We will do as suggested.

**Other Manuscript Changes**

1. We change corresponding authors from *“Chutithep Teekaput, MD”* to

*“Corresponding Author: Chatree Chai-Adisaksopha, MD, PhD*

*Division of Hematology, Department of Internal Medicine, Faculty of Medicine, Chiang Mai*

*University, Chiang Mai, 50200, Thailand*

*Tel: +66 (0) 5393 5899*

*Email: [chatree.chai@cmu.ac.th](mailto:chatree.chai@cmu.ac.th) “*
