# Supplementary material for: Outcomes of asymptomatic recombinant tissue plasminogen activator associated intracranial hemorrhage
Source: PLoS One. 2022 Aug 1;17(8):e0272257. doi: 10.1371/journal.pone.0272257 (PMC9342748; doi:10.1371/journal.pone.0272257)
Supplement: Attachment — Submitted filename: responses to reviewers_2ndrevision.docx [file pone.0272257.s003.docx]

**Reviewer Comments, Responses, and Manuscript Changes**

**Reviewer 2**

**1. For Line 140: Remove the comma before mRS.**

**“Functional outcomes, including, mRS, NIHSS, and Barthel Index were collect at the time of stroke diagnosis, 14 days, and 90 days post-rtPA.”**

Modification: We remove the comma as the reviewer suggested.

See line 327 page 7

**2. For Line 142: Remove the “a” before 90-day mRS**

**“A primary outcome of this study was a 90-day mRS.”**

Modification: We remove the “a” as the reviewer suggested.

See line 329 page 7

**3. For Line 177: Substitute ICH to sICH**

**“When comparing between patients who did not have ICH and those with aICH.”**

Modification: We deleted the sentences “When comparing between patients who did not have ICH and those with aICH, the no ICH group had more atherosclerotic and less cardioembolic subtypes per the TOAST classification. Initial presentation of patients in no ICH group was more favorable compared to aICH (lower admission NIHSS (median 9 [IQR 5, 15] vs 13 [IQR 8, 18], P-value =0.05) and higher Barthel index (median 25 [IQR 10, 55] vs 20 [IQR 5, 40], P-value =0.03)), respectively.”

These sentences were forementioned in the previous paragraph.

See line 385 page 8

**4. For Line 174-176 : As compared to the no ICH group, the aICH and sICH groups had more unfavorable admission characteristics (higher mRS, higher NIHSS and lower Barthel index).**

**The meaning is similar to line 179-181 “Initial presentation of patients in no ICH group was more favorable compared to aICH (lower admission NIHSS (median 9 [IQR 5, 15] vs 13 [IQR 8, 18], P-value =0.05) and higher Barthel index (median 25 [IQR 10, 55] vs 20 [IQR 5, 40], P-value =0.03)), “. Please combine these two parts.**

Modification: We combined the two paragraph. We deleted “Initial presentation of patients in no ICH group was more favorable compared to aICH (lower admission NIHSS (median 9 [IQR 5, 15] vs 13 [IQR 8, 18], P-value =0.05) and higher Barthel index (median 25 [IQR 10, 55] vs 20 [IQR 5, 40], P-value =0.03))”

See line 308 page 8

**5. After line 204, description regarding that bleeding progression is more common in sICH group may be added.**

**Modification:** We added the sentence “According to the lower incidence of bleeding progression in the aICH group, patients with aICH received fewer blood products than patients with sICH (7.8% vs 80.0 percent, P-value <0.001).”

See line 431 page 9

**6. For Line 216: may add “group” after sICH.**

**“Figure 2 demonstrated the 90-day mRS among no sICH, aICH, and sICH.”**

Modification: We added the word “group.”

See line 515 page 10

**7. For section after Line 214: I suggest to combine the “The 90-day modified rankin scales” with the “Outcomes of rtPA associated intracranial hemorrhage”and “Mortality”.**

Modification: We combine the “The 90-day modified rankin scales” with the “Outcomes of rtPA associated intracranial hemorrhage”and “Mortality”.

See line 526 page 10

**8. May revise the conclusion in the abstract and manuscript. May consider the below description: “rtPA associated aICH still increased the risk of morbidity and mortality outcomes. Further treatment consensus or guideline generation or clinical trials focusing on the treatment of rtPA-associated aICH may be needed”.**

Modification: We modified the sentences from “Majority of aICH patients were left untreated. Further clinical trials focusing on the treatment of rtPA-associated aICH are urgently needed in oder to improve clinical outcomes in these patients.”

to

“The rtPA associated aICH increased the risk of morbidity and mortality outcomes. Further treatment consensus, guideline generation, or clinical trials focusing on the treatment of rtPA-associated aICH may be required.”

See line 83 page 4 and line 989 page 15

**9. Please consider English editing for the grammar again.**

Comments: The manuscript was revised and language edited by English speaking person.

**Other modification**

1. In the introduction, we added the sentence “Although numerous research has focused on the long-term outcomes of sICH, there is insufficient evidence on the long-term outcomes of asymptomatic ICH (aICH). ”

See line 120 page 5

2. In the introduction, we change the sentence “This study aimed to investigate the incidence, risk factors, and characteristics of aICH secondary from thrombolytic therapy in patients who had an acute ischemic stroke. We sought to determine reversal strategies, surgical intervention, and the outcomes.” to

“The objective of this study is to determine the incidence, risk factors, and characteristics of aICH in stroke patients after receiving thrombolytic therapy. We also determine the reversal strategies, surgical intervention, and the outcomes.”

See line 124 page 5

3. In method, we change the paragraph from “Data were retrieved from The Chiang Mai University Hospital Stroke Registry between 1995 and 2019. This registry prospectively collected consecutive patients who were diagnosed with all types of acute stroke. The clinical data, laboratory, imaging, and outcomes were systematically collected. Patients with acute ischemic stroke with onset less than 3-4.5 hours, without contraindications to rtPA administration (e.g. history of previous intracranial hemorrhage or abnormal laboratory, etc.) would be given rtPA 0.9 mg/kg, maximum dose 90 mg over 60 minutes intravenously within 60 minutes after arrival at an emergency department. After that, all patients were admitted to the Acute Stroke Unit (ASU) with vital signs and neurological outcomes monitored, including the National Institute of Health Stroke Scale (NIHSS), modified Rankin scale (mRS), and Barthel index. Twenty-four hours after rtPA infusion, patients subsequently underwent a follow-up computed tomography (CT) scan of the brain to detect radiologic evidence of intracranial hemorrhage. In case of worsening of neurological symptoms, defined as an increase in NIHSS ≥ 4, patients underwent emergency brain imaging. If intracranial bleeding was detected, the comprehensive ASU care team, consisting of neurologists, neurosurgeons, rehabilitation physicians, nurses, and pharmacists evaluated and classified patients into a symptomatic or asymptomatic intracranial hemorrhage. Neurosurgical intervention or reversal agents would be given according to the comprehensive ASU care team consensus. When patients were discharged from the ASU, NIHSS, mRS, Barthel index and all-cause mortality were recorded. The NIHSS, mRS, Barthel index, and all-cause mortality would be collected again at the 14-day and 90-day follow-up visits.” to

“Data were retrieved from the Chiang Mai University Hospital Stroke Registry between 1995 and 2019. This registry prospectively collected consecutive patients who were diagnosed with all types of acute stroke. All stroke patients who were 18 years or older, diagnosed with acute ischemic stroke with onset less than 3-4.5 hours, without contraindications to rtPA administration (e.g. history of previous intracranial hemorrhage or abnormal laboratory, etc.) would be given rtPA 0.9 mg/kg, maximum dose 90 mg over 60 minutes intravenously within 60 minutes after arrival at an emergency department. The clinical data, laboratory, imaging, and outcomes were systematically collected. All patients were subsequently admitted to the Acute Stroke Unit (ASU). Vital signs and neurological outcomes including the National Institute of Health Stroke Scale (NIHSS), modified Rankin scale (mRS), and Barthel index were monitored. All patients underwent a follow-up computed tomography (CT) scan of the brain 24 hours after receiving rtPA to detect the radiologic signs of intracranial bleeding. In case of worsening neurological symptoms, defined as an increase of NIHSS ≥ 4, the patients would undergo emergency brain imaging. If intracranial bleeding was detected, the comprehensive ASU care team comprised of neurologists, neurosurgeons, rehabilitation physicians, nurses, and pharmacists would evaluate and classify patients into symptomatic or asymptomatic intracranial hemorrhage. Neurosurgical intervention or reversal agents would be given according to the comprehensive ASU care team consensus. When patients were discharged from the ASU, NIHSS, mRS, Barthel index and all-cause mortality were recorded. The NIHSS, mRS, Barthel index, and all-cause mortality would be re-collected at the 14-day and 90-day follow-up visits. Patients who did not have a follow-up brain imaging or who had missing data on functional outcomes at any time point were excluded from the study.”

See line 128 page 5
